# Sphingosine kinase 1 inhibition aggravates vascular smooth muscle cell calcification

**DOI:** 10.1007/s00424-025-03068-6

**Published:** 2025-02-03

**Authors:** Mehdi Razazian, Sheyda Bahiraii, Isratul Jannat, Adéla Tiffner, Georg Beilhack, Bodo Levkau, Jakob Voelkl, Ioana Alesutan

**Affiliations:** 1 Institute for Physiology and Pathophysiology, Johannes Kepler University Linz, Krankenhausstrasse 5, 4020 Linz, Austria; 2 Institute of Biophysics, Johannes Kepler University Linz, Linz, Austria; 3 Division of Nephrology and Dialysis, Department of Medicine III, Medical University of Vienna, Vienna, Austria; 4 Institute of Molecular Medicine III, University Hospital and Heinrich Heine University Düsseldorf, Düsseldorf, Germany; 5 Department of Nephrology and Medical Intensive Care, Charité-Universitätsmedizin Berlin, Berlin, Germany; 6 DZHK (German Centre for Cardiovascular Research), Partner Site Berlin, Berlin, Germany

**Keywords:** Sphingosine kinase 1, Phosphate, Vascular smooth muscle cells, Vascular calcification

## Abstract

Medial vascular calcification is common in chronic kidney disease patients and linked to hyperphosphatemia. Upon phosphate exposure, intricate signaling events orchestrate pro-calcific effects in the vasculature mediated by vascular smooth muscle cells (VSMCs). Sphingosine kinase 1 (SPHK1) produces sphingosine-1-phosphate (S1P) and is associated with complex effects in the vascular system. The present study investigated a possible involvement of SPHK1 in VSMC calcification. Experiments were performed in primary human aortic VSMCs under pro-calcific conditions, with pharmacological inhibition or knockdown of SPHK1 or SPNS2 (a lysolipid transporter involved in cellular S1P export), as well as in Sphk1-deficient and wild-type mice treated with cholecalciferol. In VSMCs, SPHK1 expression was up-regulated by pro-calcific conditions. Calcification medium up-regulated osteogenic marker mRNA expression and activity as well as calcification of VSMCs, effects significantly augmented by co-treatment with the SPHK1 inhibitor SK1-IN-1. SK1-IN-1 alone was sufficient to up-regulate osteogenic signaling in VSMCs during control conditions. Similarly, the SPHK1 inhibitor PF-543 and SPHK1 knockdown up-regulated osteogenic signaling in VSMCs and aggravated VSMC calcification. In contrast, co-treatment with the SPNS2 inhibitor SLF1081851 suppressed osteogenic signaling and calcification of VSMCs, effects abolished by silencing of SPHK1. In addition, Sphk1 deficiency aggravated vascular calcification and aortic osteogenic marker expression in mice after cholecalciferol overload. In conclusion, SPHK1 inhibition, knockdown, or deficiency aggravates vascular pro-calcific signaling and calcification. The reduced calcification after inhibition of S1P export suggests a possible involvement of intracellular S1P, but further studies are required to elucidate the complex roles of SPHKs and S1P signaling in calcifying VSMCs.

## Introduction

Medial vascular calcification (VC) is defined by the accumulation of calcium phosphate complexes in the medial arterial layer [39]. This type of calcification is distinct from intimal calcification, which is linked to atherosclerosis [39]. VC is especially associated with chronic kidney disease (CKD), as well as diabetes mellitus and aging [39, 70]. The presence of VC is an independent risk factor for mortality [39], and no broadly applicable therapeutic strategies are available.

Complex local and systemic events act in concert to promote VC [71], where an important regulatory role is attributed to vascular smooth muscle cells (VSMCs) [18]. VSMCs are able to facilitate a pro-calcific micro-environment in response to various stimuli, such as inflammatory mediators [5, 6], endothelial dysfunction, and unphysiological stress [17, 66, 76]. One of the most powerful promotors of VC is disturbed phosphate homeostasis with hyperphosphatemia in CKD [33]. The pro-calcific effects of VSMCs are mediated by various factors to directly or indirectly augment the formation of calcium phosphate crystals, such as extracellular vesicle release [58], release of inflammatory mediators [71], or degradation of the calcification inhibitor pyrophosphate by tissue-nonspecific alkaline phosphatase (ALPL) [65]. The calcific response of VSMCs is linked to the activation of pro-calcific transcription factors such as core-binding factor alpha 1 (CBFA1, also known as runt-related transcription factor 2 or RUNX2), which is required for the development of VC [41, 42]. Various inflammatory and other pathways converge on a pro-calcific gene expression profile, but the multifaceted upstream components are still incompletely defined [70].

Sphingosine-1-phosphate (S1P) is a vasoactive sphingolipid, with a complex role in vascular tone, endothelial barrier function, and lymphocyte trafficking [34]. Both intra- and extra-cellular functions of S1P are described [62]. In macrophages, S1P induces ATP efflux, which is the substrate for the formation of the anti-calcific pyrophosphate by ectonucleotide pyrophosphatase/phosphodiesterase 1 (ENPP1) [15, 67]. S1P is formed by the phosphorylation of sphingosine through sphingosine kinases (SPHKs) SPHK1 and SPHK2 [73]. S1P can be transported outside the cell through SPNS lysolipid transporter 2, S1P (SPNS2) [30]. The SPHKs differ in subcellular distribution and function [55]. SPHK1 is expressed in VSMCs, and its deficiency reduces proliferation and protects from pulmonary hypertension [16]. SPHK1 inhibition also impacts on atherosclerosis [55]. In calcifying VSMCs, S1P production is increased, where extracellular S1P promotes calcification [46]. SPHK function may also govern the balance between S1P and ceramide, which have been associated with opposite effects in apoptosis signaling [48]. The ceramide system has been linked to cardiovascular calcification. A pro-calcific role of acid sphingomyelinase [10, 43], neutral sphingomyelinase 2 [52], or ceramide synthase 5 [57] was described, while the acid ceramidase was shown to mediate anti-calcific effects [11].

In view of the complex roles attributed to SPHKs and S1P, the present study explores the putative function of SPHK1 in VSMC calcification during exposure to elevated phosphate concentrations.

## Materials and methods

### Cell culture

Primary human aortic smooth muscle cells (HAoSMCs, Fisher Scientific) [4, 5, 26] were routinely cultured in a medium containing a 1:1 ratio of Waymouth’s MB 752/1 and Ham’s F-12 nutrient mix supplemented with 10% FBS, 100 U/ml penicillin, and 100 µg/ml streptomycin (all from Fisher Scientific) and were used in experiments up to passage 12. Cells were treated for the indicated times with calcification medium (culture medium additionally supplemented with 10 mM β-glycerophosphate and 1.5 mM CaCl_2_ (Sigma Aldrich)) and/or with 10 µM SK1-IN-1 (SPHK1 inhibitor, stock in DMSO, MedChemExpress) [74], 10 µM PF-543 (SPHK1 inhibitor, stock in DMSO, MedChemExpress) [23], and 3 µM SLF1081851 (SPNS2 inhibitor, stock in ethanol, MedChemExpress) [22]. Treatment with equal amounts of vehicle were used as control. Where indicated, cells were transfected with 10 nM SPHK1 (ID: s16959) or negative control (ID: 4390843) siRNA using siPORT amine transfection reagent (all from Fisher Scientific). For long-term treatments, fresh medium with agents were added every 2–3 days.

### Animal experiments

All animal experiments were approved by the local authorities (BMBWF Vienna, Austria, 66.009/0320-V/3b/2019). The generation and origin of Sphk1-deficient mice was described previously [8]. Calcification was induced by high-dosed cholecalciferol treatment [3] in male Sphk1-deficient (sphk1^−/−^) and corresponding wild-type (sphk1^+/+^) mice. Mice were injected subcutaneously with 500,000 IU/kg BW of cholecalciferol (Sigma Aldrich) or vehicle for 3 successive days. On day 6, blood was collected by retroorbital puncture and mice were sacrificed by cervical dislocation during isoflurane anesthesia. Aortic arches (calcium content) and thoracic aorta (mRNA expression) were snap frozen for further experiments (*n* = 5). Serum concentrations of calcium and phosphate were determined by using a photometric method (FUJI Dri-Chem Nx700). For Alizarin Red staining (*n* = 4), whole aortas were carefully excised and stained with Alizarin Red (0.0016% in 0.5% KOH) [7].

### RNA isolation and RT-PCR

Total RNA was isolated by using Trizol Reagent (Fisher Scientific), and reverse transcription was performed by using SuperScript III Reverse Transcriptase and oligo(dT)_12–18_ primers (Fisher Scientific). RT-PCR was performed in duplicate with iQ Sybr Green Supermix (Bio-Rad Laboratories) and the following primers (Fisher Scientific) [69, 72]:

Human primers:

- *ALPL* fw: GGGACTGGTACTCAGACAACG
- *ALPL* rev: GTAGGCGATGTCCTTACAGCC
- *CBFA1* fw: GCCTTCCACTCTCAGTAAGAAGA
- *CBFA1* rev: GCCTGGGGTCTGAAAAAGGG
- *GAPDH* fw: GAGTCAACGGATTTGGTCGT
- *GAPDH* rev: GACAAGCTTCCCGTTCTCAG
- *SPHK1* fw: GGCTGCTGTCACCCATGAA

- *SPHK1* rev: TCACTCTCTAGGTCCACATCAG

Mouse primers:

- *Alpl* fw: TTGTGCCAGAGAAAGAGAGAGA
- *Alpl* rev: GTTTCAGGGCATTTTTCAAGGT
- *Cbfa1* fw: AGAGTCAGATTACAGATCCCAGG
- *Cbfa1* rev: AGGAGGGGTAAGACTGGTCATA
- *Gapdh* fw: AGGTCGGTGTGAACGGATTTG

- *Gapdh* rev: TGTAGACCATGTAGTTGAGGTCA

Relative mRNA fold changes were calculated by the 2^−ΔΔCt^ method using GAPDH as internal reference.

### Protein isolation and Western blotting

Total proteins were isolated by using ice-cold Pierce IP lysis buffer supplemented with complete protease and phosphatase inhibitors cocktail (all from Fisher Scientific), and protein concentration was determined by the Bradford assay (Bio-Rad Laboratories). Equal amounts of protein were boiled in Roti-Load1 Buffer (Carl Roth) at 100 °C for 10 min and then separated on SDS-PAGE gels and transferred to PVDF membranes (Roche Applied Science). Membranes were incubated with primary antibodies: rabbit anti-SPHK1 (1:1000, #12071, Cell Signaling) and rabbit anti-GAPDH (1:3000, #2118, Cell Signaling) at 4 °C overnight and with secondary anti-rabbit HRP-conjugated antibody (1:1000, Cell Signaling) at room temperature for 1 h. Membranes were stripped with Restore Plus Western blot stripping buffer (Fisher Scientific) at room temperature. Bands were detected with Clarity Western ECL substrate (Bio-Rad Laboratories) using the ChemiDoc MP imaging system (Bio-Rad Laboratories) and quantified using the ImageJ software. Data are shown as the ratio of total protein to GAPDH, normalized to the control group [28].

### Immunofluorescence staining

HAoSMCs were fixed in 4% PFA/PBS for 15 min at room temperature, permeabilized in 0.3% TritonX-100/PBS for 10 min at room temperature, and blocked with 5% goat serum in 0.1% TritonX-100/PBS for 60 min at room temperature. Cells were incubated with primary rabbit anti-RUNX2 antibody (1:100 in 0.1% TritonX-100/PBS, #12556, Cell Signaling) [7] overnight at 4 °C and then with goat anti-rabbit Alexa488-conjugated antibody (1:500 in 0.1% TritonX-100/PBS, Invitrogen) for 2 h at room temperature. Nuclei were stained with 0.5 µg/ml DAPI (Carl Roth) for 5 min at room temperature, and slides were mounted with Prolong Diamond antifade reagent (Invitrogen). Images were acquired on a Nikon Ti-2 microscope (× 60 oil immersion, NA 1.42) equipped with a Clarity Laser Free Confocal Unit (Aurox).

### ALP activity

ALP activity in cell lysates was determined by using a colorimetric ALP assay kit (Abcam) and protein concentration was determined by the Bradford assay (Bio-Rad Laboratories). Data are shown normalized to total protein concentration and to the control group [6].

### Calcification staining

At the end of the treatment period, cells were incubated with OsteoSense 680EX (1:250, Perkin Elmer) overnight at 37 °C, and images were acquired with the ChemiDoc MP imaging system (Bio-Rad Laboratories) [53].

### Quantification of calcium content

Cells and aortic tissues were decalcified in 0.6 M HCl overnight at 4 °C and 37 °C, respectively. The calcium content in the supernatant was quantified by using the QuantiChrom Calcium assay kit (BioAssay Systems). Proteins were isolated by using 0.1 M NaOH/ 0.1% SDS buffer and quantified by the Bradford assay (Bio-Rad Laboratories). Data are shown normalized to total protein concentration and to the control group [47].

### Statistics

Data are shown as scatter dot plots and arithmetic means ± SEM, and* n* represents the number of independent experiments performed. Normalized data are presented as arbitrary units (a.u.). Normality was determined by the Shapiro–Wilk test. For multiple group comparison, statistical testing was performed by using one-way ANOVA with Tukey’s test (homoscedastic data) or Games-Howell test (heteroscedastic data) and Kruskal–Wallis with Steel–Dwass test (non-normal data). For two groups, statistical testing was performed using *T*-test. *p* < 0.05 was considered statistically significant.

## Results

**Fig. 1:**
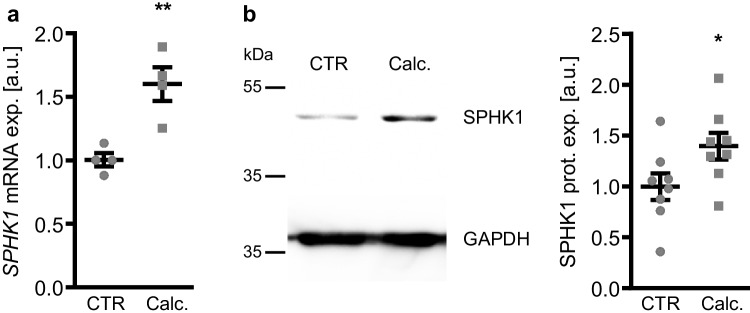
SPHK1 expression was up-regulated in VSMCs during pro-calcific conditions. **a** Relative mRNA expression (*n* = 4) of *SPHK1* in HAoSMCs treated for 24 h with control (CTR) or calcification medium (Calc.). **b** Representative Western blots and normalized SPHK1 protein abundance (*n* = 8) in HAoSMCs treated for 24 h with control (CTR) or calcification medium (Calc.). *(*p* < 0.05), **(*p* < 0.01) significant vs. control group

To investigate whether SPHK1 may play a role in VSMC calcification during pro-calcific conditions, a first series of experiments was performed in primary HAoSMCs following exposure to control medium or calcification medium supplemented with calcium and the phosphate donor β-glycerophosphate. As a result, *SPHK1* mRNA expression (Fig. 1a) and protein abundance (Fig. 1b) were significantly higher in HAoSMCs treated for 24 h with calcification medium as compared to control HAoSMCs.

**Fig. 2:**
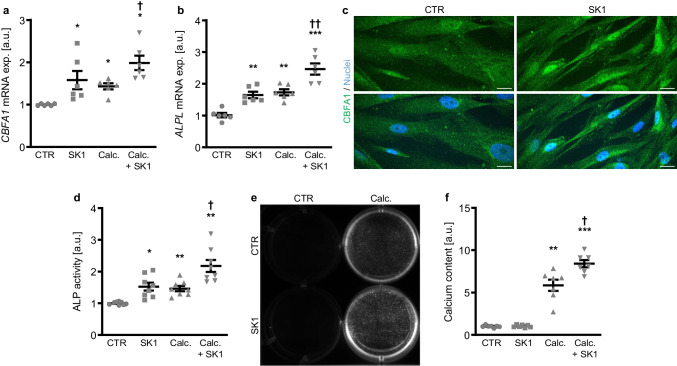
The SPHK1 inhibitor SK1-IN-1 augmented osteogenic marker expression and activity as well as calcification of VSMCs during pro-calcific conditions. Relative mRNA expression (*n* = 6) of *CBFA1* (**a**) and *ALPL* (**b**) in HAoSMCs treated for 48 h with control (CTR) or calcification medium (Calc.) without and with 10 µM SK1-IN-1 (SK1). **c** CBFA1 (green) and nuclei (blue) shown by confocal imaging in HAoSMCs treated for 48 h with control (CTR) or 10 µM SK1-IN-1 (SK1). Scale bar: 20 µm. **d** Normalized ALP activity (*n* = 8) in HAoSMCs treated for 7 days with control (CTR) or calcification medium (Calc.) without and with 10 µM SK1-IN-1 (SK1). Calcification detected by Osteosense fluorescence imaging (**e**) and normalized calcium content (**f**) (*n* = 7) in HAoSMCs treated for 11 days with control (CTR) or calcification medium (Calc.) without and with 10 µM SK1-IN-1 (SK1). Calcified areas: white pseudocolor. *(*p* < 0.05), **(*p* < 0.01), ***(*p* < 0.001) significant vs. control group; †(*p* < 0.05), ††(*p* < 0.01) significant vs. Calc.-treated group

Next, the effects of SPHK1 suppression using pharmacological inhibitors on calcification medium-induced osteogenic signaling and VSMC calcification were determined. As shown in Fig. 2a, b, calcification medium induced up-regulated mRNA expression of the osteogenic markers *CBFA1* and *ALPL*, effects significantly augmented in the presence of the SPHK1 inhibitor SK1-IN-1. Treatment with SK1-IN-1 alone was sufficient to up-regulate osteogenic marker expression in HAoSMCs. As shown by immunostaining and confocal microscopy, SK1-IN-1 increased CBFA1 nuclear localization in HAoSMCs (Fig. 2c). In addition, SK1-IN-1 significantly increased ALP activity and augmented calcification medium-induced ALP activity in HAoSMCs (Fig. 2d). More importantly, as illustrated by Osteosense fluorescence imaging and quantification of calcium content, SK1-IN-1 did not significantly modify calcification of HAoSMCs during control conditions, but significantly aggravated HAoSMC calcification induced by the calcification medium (Fig. 2e, f).

**Fig. 3:**
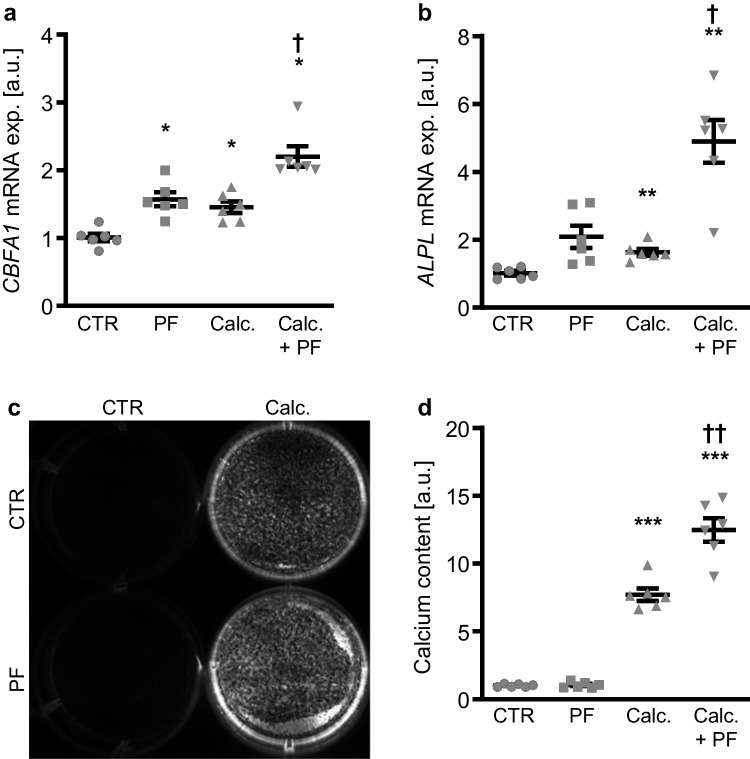
The SPHK1 inhibitor PF-543 augmented osteogenic marker expression and calcification of VSMCs during pro-calcific conditions. Relative mRNA expression (*n* = 6) of *CBFA1* (**a**) and *ALPL* (**b**) in HAoSMCs treated for 48 h with control (CTR) or calcification medium (Calc.) without and with 10 µM PF-543 (PF). Calcification detected by Osteosense fluorescence imaging (**c**) and normalized calcium content (**d**) (*n* = 6) in HAoSMCs treated for 11 days with control (CTR) or calcification medium (Calc.) without and with 10 µM PF-543 (PF). Calcified areas: white pseudocolor. *(*p* < 0.05), **(*p* < 0.01), ***(*p* < 0.001) significant vs. control group; †(*p* < 0.05), ††(*p* < 0.01) significant vs. Calc.-treated group

Similarly, the SPHK1 inhibitor PF-543 significantly increased the mRNA expression of *CBFA1* and tended to up-regulate *ALPL* mRNA expression, a difference, however, not reaching statistical significance (*p* = 0.075), in HAoSMCs during control conditions (Fig. 3a, b). Furthermore, PF-543 significantly augmented calcification medium-induced osteogenic markers mRNA expression as well as calcification of HAoSMCs (Fig. 3a–d). Taken together, SPHK1 inhibition promoted osteogenic signaling in VSMCs and augmented osteogenic marker expression and calcification of VSMCs during pro-calcific conditions.

**Fig. 4:**
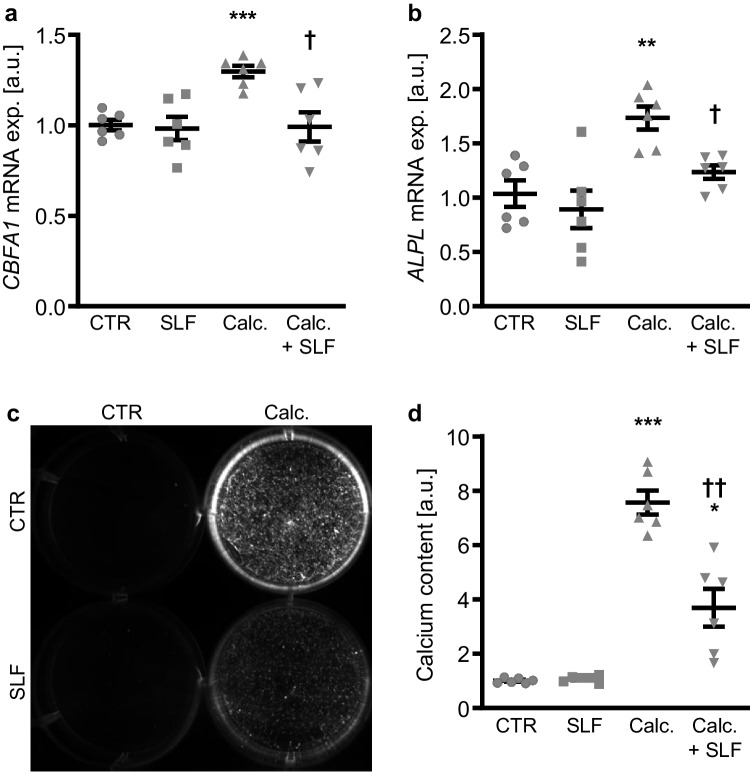
The SPNS2 inhibitor SLF1081851 reduced osteogenic marker expression and calcification of VSMCs during pro-calcific conditions. Relative mRNA expression (*n* = 6) of *CBFA1* (**a**) and *ALPL* (**b**) in HAoSMCs treated for 48 h with control (CTR) or calcification medium (Calc.) without and with 3 µM SLF1081851 (SLF). Calcification detected by Osteosense fluorescence imaging (**c**) and normalized calcium content (**d**) (*n* = 6) in HAoSMCs treated for 11 days with control (CTR) or calcification medium (Calc.) without and with 3 µM SLF1081851 (SLF). Calcified areas: white pseudocolor. *(*p* < 0.05), **(*p* < 0.01), ***(*p* < 0.001) significant vs. control group; †(*p* < 0.05), ††(*p* < 0.01) significant vs. Calc.-treated group

In further experiments, the effects of SPNS2 transporter inhibition by using SLF1081851 to block S1P export on osteogenic signaling and VSMC calcification during pro-calcific conditions were investigated. Co-treatment with SLF1081851 significantly suppressed osteogenic marker expression induced by calcification medium in HAoSMCs (Fig. 4a, b). In addition, SLF1081851 significantly reduced the calcification of HAoSMCs during pro-calcific conditions (Fig. 4c, d). Thus, blockage of S1P export via SPNS2 was able to ameliorate osteogenic signaling and calcification of VSMCs during pro-calcific conditions.

**Fig. 5:**
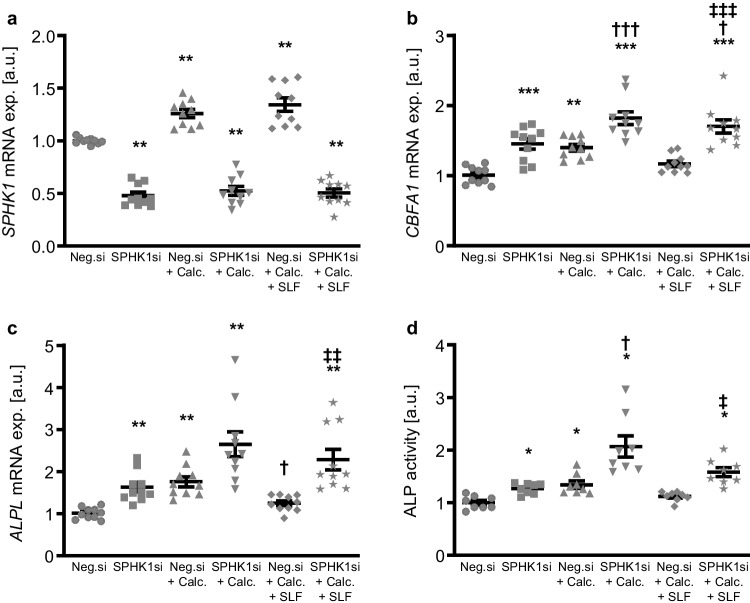
SPHK1 knockdown augmented osteogenic marker expression and activity and suppressed the protective effects of SPNS2 inhibition during pro-calcific conditions. Relative mRNA expression (*n* = 10) of *SPHK1* (**a**), *CBFA1* (**b**), and *ALPL* (**c**) in HAoSMCs transfected for 72 h with negative control siRNA (Neg.si) or SPHK1 siRNA (SPHK1si) and treated for 48 h with control or calcification medium (Calc.) without and with 3 µM SLF1081851 (SLF). **d** Normalized ALP activity (*n* = 8) in HAoSMCs transfected with negative control siRNA (Neg.si) or SPHK1 siRNA (SPHK1si) and treated for 7 days with control or calcification medium (Calc.) without and with 3 µM SLF1081851 (SLF). *(*p* < 0.05), **(*p* < 0.01), ***(*p* < 0.001) significant vs. Neg.si-transfected group; †(*p* < 0.05), †††(*p* < 0.001) significant vs. Neg.si + Calc.-treated group; ‡(*p* < 0.05), ‡‡(*p* < 0.01), ‡‡‡(*p* < 0.001) significant between Neg.si + Calc. + SLF- and SPHK1si + Calc. + SLF-treated groups

**Fig. 6:**
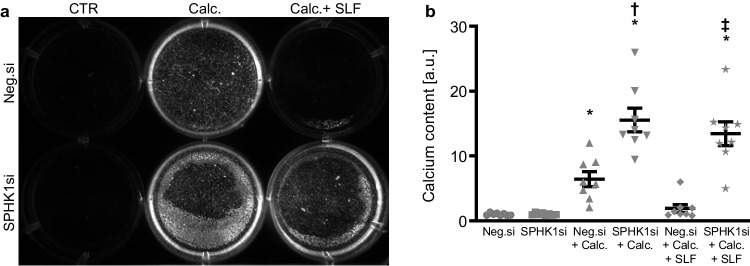
SPHK1 knockdown aggravated VSMC calcification and abolished the protective effects of SPNS2 inhibition during pro-calcific conditions. Calcification detected by Osteosense fluorescence imaging (**a**) and normalized calcium content (**b**) (*n* = 8) in HAoSMCs transfected with negative control siRNA (Neg.si) or SPHK1 siRNA (SPHK1si) and treated for 11 days with control (CTR) or calcification medium (Calc.) without and with 3 µM SLF1081851 (SLF). Calcified areas: white pseudocolor. *(*p* < 0.05) significant vs. Neg.si-transfected group; †(*p* < 0.05) significant vs. Neg.si + Calc.-treated group; ‡(p < 0.05) significant between Neg.si + Calc. + SLF- and SPHK1si + Calc. + SLF-treated groups

In the next series of experiments, the endogenous expression of SPHK1 was suppressed by using small interfering RNA (siRNA) in HAoSMCs exposed to control or calcification medium. To indicate a role of intracellular S1P during SPHK1 knockdown, experiments were conducted in the presence and absence of the SPNS2 inhibitor SLF1081851. As a result, transfection with SPHK1 siRNA significantly reduced *SPHK1* mRNA expression in HAoSMCs as compared to negative control siRNA-transfected HAoSMCs (Fig. 5a). In accordance with our previous finding, exposure to calcification medium significantly increased *SPHK1* mRNA expression, an effect not significantly modified by co-treatment with SLF1081851. Knockdown of SPHK1 significantly up-regulated *CBFA1* and *ALPL* mRNA expression as well as ALP activity in HAoSMCs (Fig. 5b–d) and significantly augmented calcification medium-induced *CBFA1* mRNA expression and ALP activity and tended to increase calcification medium-induced *ALPL* mRNA expression (*p* = 0.095). Furthermore, silencing of SPHK1 significantly reversed the protective effects of SLF1081851 on osteogenic marker expression and ALP activity in HAoSMCs. In accordance, silencing of SPHK1 aggravated HAoSMC calcification and abolished the anti-calcific effects of SLF1081851 during pro-calcific conditions (Fig. 6). Taken together, knockdown of SPHK1 promoted osteogenic signaling and augmented calcification of VSMCs during pro-calcific conditions. In addition, blockage of S1P export via SPNS2 failed to reduce osteogenic signaling and calcification of SPHK1-silenced VSMCs during pro-calcific conditions.

**Fig. 7:**
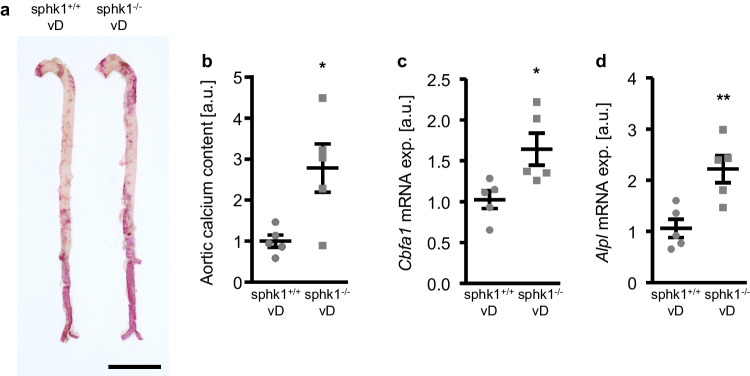
Sphk1 deficiency aggravated vascular calcification in mice during cholecalciferol overload. **a** Alizarin Red staining of aortas from Sphk1-deficient (sphk1^−/−^) and corresponding wild-type (sphk1^+/+^) mice receiving high-dosed cholecalciferol (vD). Calcification: red staining; scale bar: 5 mm. Normalized calcium content (**b**) as well as relative mRNA expression of *Cbfa1* (**c**) and *Alpl* (**d**) (*n* = 5) in aortic tissue from Sphk1-deficient (sphk1^−/−^) and corresponding wild-type (sphk1^+/+^) mice receiving high-dosed cholecalciferol (vD). *(*p* < 0.05), **(*p* < 0.01) significant vs sphk1^+/+^ vD-treated group

To confirm the in vitro findings, additional experiments were performed in vivo in the cholecalciferol overload-induced VC mouse model using the Sphk1-deficient (sphk1^−/−^) and corresponding wild-type (sphk1^+/+^) mice. As shown in Table 1, the serum calcium and phosphate levels were similar in both genotypes treated with high-dosed cholecalciferol. More importantly, as shown by Alizarin Red staining and quantification of calcium content, the aortic calcification was significantly higher in the cholecalciferol-treated sphk1^−/−^ mice as compared to sphk1^+/+^ mice (Fig. 7a, b). In accordance, the aortic osteogenic marker *Cbfa1* and *Alpl* mRNA expression were significantly higher in the cholecalciferol-treated sphk1^−/−^ mice as compared to sphk1^+/+^ mice (Fig. 7c, d). Thus, Sphk1 deficiency aggravated VC in mice during cholecalciferol overload.

**Table 1:** Effects of Sphk1 deficiency in mice during cholecalciferol overload. Serum calcium and phosphate levels in Sphk1-deficient (sphk1^−/−^) and corresponding wild-type (sphk1^+/+^) mice receiving high-dosed cholecalciferol (vD)

	sphk1^+/+^ vD	sphk1^−/−^ vD	*p*-value	
Calcium [mg/dl]	19.56 ± 1.02	21.00 ± 0.78	0.295	*n* = 5
Phosphate [mg/dl]	8.14 ± 0.17	7.92 ± 0.57	0.719	*n* = 5

## Discussion

The present study discloses a novel role of SPHK1 in the signaling of VSMC calcification in vitro and in vivo. Pharmacological inhibition, knockdown, or deficiency of SPHK1 augments VSMC calcification. Moreover, inhibition of S1P export via SPNS2 is protective during VSMC calcification in vitro.

These findings add a further aspect to the complex role of SPHKs in cardiovascular pathologies. Up-regulation of SPHK1 is observed in VSMCs during calcifying conditions, as was also reported previously in bovine VSMCs [46]. Platelet-derived growth factor (PDGF), a potent stimulator of VSMC calcification [68], increases SPHK1 expression in VSMCs [21]. Increased SPHK1 expression is also observed in calcified aortic valves [9]. In theory, up-regulation of SPHK1 could be a general response to stressed conditions and pro-apoptotic signaling [24], which could also link to pro-calcific signaling [17]. But the current findings are in some contrast to known functions of SPHK1 in cardiovascular pathologies and its attributed pro-inflammatory function [14]. Some discrepant findings were observed in atherosclerosis development where SPHK2 inhibition by ABC294640 induces complex alterations and ultimately does not affect atherosclerosis [54]. Nonetheless, aggravated atherosclerosis is observed in Sphk2- and not Sphk1-deficient mice [32]. Sphk1 overexpression in Sphk2-deficient mice rescues aggravated atherosclerosis [32]. Sphk1 inhibition with SKI-II exacerbates atherosclerosis in mice [55]. Increasing S1P levels reduces atherosclerosis development, but its effect may depend on the S1P receptor involved and other factors [20]. In contrast, pharmacological inhibition of S1P degradation by S1P lyase aggravates atherosclerosis [37]. Adding further complexity, hypoxia-induced pulmonary hypertension is ameliorated by SPHK1 deficiency [16]. However, silencing of SPHK1 in pulmonary VSMCs reduces the proliferative effect of PDGF [40]. Despite aggravated endothelial dysfunction, the pro-calcific mediator angiotensin II [71] has a reduced hypertensive effect in Sphk1-deficient mice [61]. S1P maintains endothelial barrier function and induces vascular nitric oxide generation [36], which has important anti-calcific effects on VSMCs [3]. The expression of SPHK1 also differs among different sections of the arterial tree [59]. The function of SPHK1 in the vasculature appears therefore rather complex and context-dependent.

The diverse functional consequences of SPHK1 activation could theoretically involve differing effects of intracellular and extracellular S1P. Extracellular S1P acts through G protein-coupled receptors in the plasma membrane [38]. Extracellular S1P has been shown to aggravate calcification of valvular interstitial cells through S1P receptor 2 [9]. In bovine VSMCs, the addition of exogenous S1P aggravates calcification [46]. Seemingly in contrast, we observe increased VSMC calcification upon SPHK1 inhibition. However, VSMC calcification appears to be reduced by inhibition of SPNS2-mediated S1P export, which is ineffective during conditions of SPHK1 knockdown. This indicates that anti-calcific effects of S1P may be mediated by its intracellular second messenger function and independent of the cell surface receptors. The importance of cellular S1P export is supported by the observation that blockade of SPNS2-mediated S1P export ameliorates renal fibrosis [64]. In HK2 cells, SPHK1 silencing and SPNS2 overexpression augments the expression of connective tissue growth factor (CTGF), while SPNS2 silencing reduces it [12]. CTGF itself has been linked to pro-calcific effects [31]. Also, deficiency of a cardiac S1P transporter induces protection against angiotensin-II-induced cardiac remodelling, an effect attributed to elevated intracellular S1P [19]. The importance of intracellular S1P in VSMCs is also indicated by the observation that intracellular S1P appears to be required for the anti-apoptotic effects of SPHK1 during high glucose conditions [77]. Similarly, SPHK1 governs the induction of VSMC mitogenesis by factor-Xa [13], which is able to induce VSMC senescence in atherosclerotic plaques [60]. Activation of apoptotic and senescence pathways are considered hallmarks of pro-calcific signaling in VSMCs [56]. Thus, deranged intracellular S1P levels could be responsible for the aggravated calcification during SPHK1 inhibition.

However, the pathways of intracellular second messenger functions of S1P produced by SPHKs are less well defined [62]. S1P regulates lysosomal function, homeostasis, and lysosome-associated membrane proteins [75]. Intracellular accumulation of S1P impairs lysosomal function [35], which is essential for phosphate-induced VSMC calcification [2]. Other possible intracellular targets of S1P include histone deacetylases [25], TNF receptor-associated factor 2 (TRAF2) [50], baculoviral IAP repeat-containing protein3 (cIAP2) [27], mitochondrial prohibitin 2 [63], peroxisome proliferator-activated receptor (PPAR) γ [49], or heat shock proteins: heat shock protein 90 alpha family class A member 1 (HSP90α) and glucose-regulated protein 94 (GRP94) [51].

With the complex and incompletely defined intracellular signaling effects of S1P, the exact mechanisms underlying the pro-calcific effects of SPHK1 inhibition in VSMCs, contrasting other models [9], can currently not be clearly delineated. Compared to other cell types, it could be hypothesized that VSMCs are more sensitive to intracellular S1P concentrations and the finely tuned balance of signaling molecules within this pathway. Nonetheless, other mechanisms may be of relevance for the effects of SPHK1 inhibition on VC. The effects on calcification might not be directly mediated by SPHK1, as SPHK1 and SPHK2 have diverse and sometimes opposing functions [62]. But some compensatory substitution between SPHK1 and SPHK2 seems apparent. Both Sphk1- or Sphk2-deficient mice are viable, and only their double knockout leads to embryonic lethality [45]. A hypothetical compensatory shift to increased SPHK2 activity during SPHK1 inhibition or deficiency could play a role in the current observation. Furthermore, the effect of SPHK1 on calcification may not be solely mediated by S1P, as SPHK1 decreases ceramide levels, while SPHK2 may increase these [44]. Increased ceramide accumulation in VSMC during SPHK1 inhibition could induce pro-calcific effects [10, 43]. Also, elevated intracellular S1P could lead to its increased intracellular degradation, where sphingosine phosphate lyase ultimately yields ethanolamine phosphate and hexadecenal [1]. These degradation products themselves could exert important biological effects [1]. The current study is thereby limited, as it did not determine the concentrations of S1P and its degradation products. Also, it must be kept in mind that the effects due to modifying the finely tuned balance of S1P signaling systems could be sensitive to the experimental models used and the timepoints investigated. The cell culture experiments might not completely reflect pathological processes in the human CKD patient. Also, cholecalciferol treatment induces rapid calcification in mice but differs to the calcifying conditions typically observed in CKD patients [29]. Therefore, the animal experiments might be biased by the model used and sex-dependent or CKD-specific effects cannot be interpreted. Thus, the interpretations from the current observations are limited and cannot be generalized, especially towards the human patient.

**Fig. 8:**
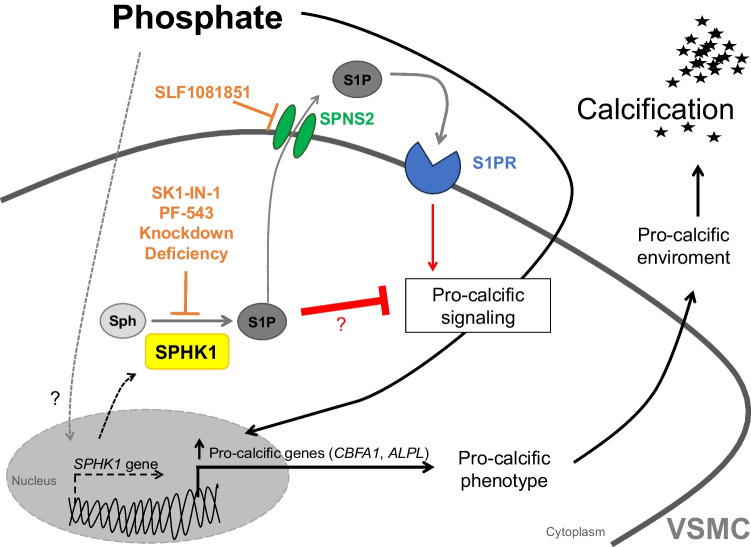
Hypothesis on the impact of SPHK1 inhibition on VSMC calcification. Chronic kidney disease induces hyperphosphatemia, which trippers complex pro-calcific signaling pathways in vascular smooth muscle cells (VSMCs), fostering a pro-calcific micro-environment. Phosphate also up-regulates sphingosine kinase 1 (SPHK1) expression in VSMCs. Cellular SPHK1 mediates the synthesis of sphingosine-1-phosphate (S1P) by phosphorylation of sphingosine (Sph). Cytoplasmic S1P can be exported by sphingolipid transporter 2 (SPNS2). Extracellular S1P can induce pro-calcific effects [9, 46], while intracellular S1P mediates anti-calcific effects by unknown mechanisms. In calcifying VSMCs, the anti-calcific effects of intracellular S1P might outweigh the pro-calcific effects of extracellular S1P, as (a) pharmacological inhibition (SK1-IN-1, PF-543), knockdown, or deficiency of SPHK1 increase pro-calcific signaling, (b) blockade of S1P export by pharmacological SPNS2 inhibition (SLF1081851) ameliorates pro-calcific signaling, and (c) the anti-calcific effects of SPNS2 inhibition are blocked during SPHK1 knockdown. However, further evidence is required to support this hypothesis and other factors or mechanisms not depicted may be involved

In conclusion, SPHK1 inhibitors or SPHK1 knockdown aggravate, while inhibition of S1P export ameliorates calcification in VSMCs (Fig. 8). Sphk1 deficiency increases VC in mice after cholecalciferol treatment. These observations hint at an important function of intracellular S1P generated by SPHK1 during VC (Fig. 8), but further studies are required to pinpoint the underlying mechanisms and the translational relevance.

## Data Availability

No datasets were generated or analysed during the current study.
